# Utilizing COVID-19 as a Model for Diagnostics Using an Electrochemical Sensor

**DOI:** 10.3390/s24123772

**Published:** 2024-06-10

**Authors:** Ava Gevaerd, Emmanuelle A. Carneiro, Jeferson L. Gogola, Diego R. P. Nicollete, Erika B. Santiago, Halanna P. Riedi, Adriano Timm, João V. Predebon, Luis F. Hartmann, Victor H. A. Ribeiro, Carlos Rochitti, Gustavo L. Marques, Maira M. O. N. Loesch, Bernardo M. M. de Almeida, Sérgio Rogal-Junior, Marcus V. M. Figueredo

**Affiliations:** 1 Research and Development Department, Hilab Campus, Rua José A. Possebom, 800, Curitiba, Parana 81270-185, Brazilmarcus@hilab.com.br (M.V.M.F.); 2 School of Medicine—Campus PUCPR, Rua Imaculada Conceição, 1155, Prado Velho, Curitiba, Parana 80215-901, Brazil

**Keywords:** SARS-CoV-2, nucleocapsid protein, point-of-care test, electrochemical sensor, screen-printed electrodes

## Abstract

This paper reports a rapid and sensitive sensor for the detection and quantification of the COVID-19 N-protein (N-PROT) via an electrochemical mechanism. Single-frequency electrochemical impedance spectroscopy was used as a transduction method for real-time measurement of the N-PROT in an immunosensor system based on gold-conjugate-modified carbon screen-printed electrodes (Cov-Ag-SPE). The system presents high selectivity attained through an optimal stimulation signal composed of a 0.0 V DC potential and 10 mV RMS^−1^ AC signal at 100 Hz over 300 s. The Cov-Ag-SPE showed a log response toward N-PROT detection at concentrations from 1.0 ng mL^−1^ to 10.0 μg mL^−1^, with a 0.977 correlation coefficient for the phase (θ) variation. An ML-based approach could be created using some aspects observed from the positive and negative samples; hence, it was possible to classify 252 samples, reaching 83.0, 96.2 and 91.3% sensitivity, specificity, and accuracy, respectively, with confidence intervals (CI) ranging from 73.0 to 100.0%. Because impedance spectroscopy measurements can be performed with low-cost portable instruments, the immunosensor proposed here can be applied in point-of-care diagnostics for mass testing, even in places with limited resources, as an alternative to the common diagnostics methods.

## 1. Introduction

Effective rapid-testing and point-of-care (POC) devices are a simplified, easy-to-operate, and decentralized diagnostic alternative that can measure critical physiological parameters, providing data that allow rapid and effective diagnostics [1]. These tests represent an important tool for diagnostic medicine, as in addition to speeding up medical diagnosis, they have low overall costs compared to equipment used by conventional laboratories. Therefore, the search for detection platforms with high accuracy and, above all, high portability has been the driving force behind many studies within the scientific community [2,3]. 

**Figure 1:**
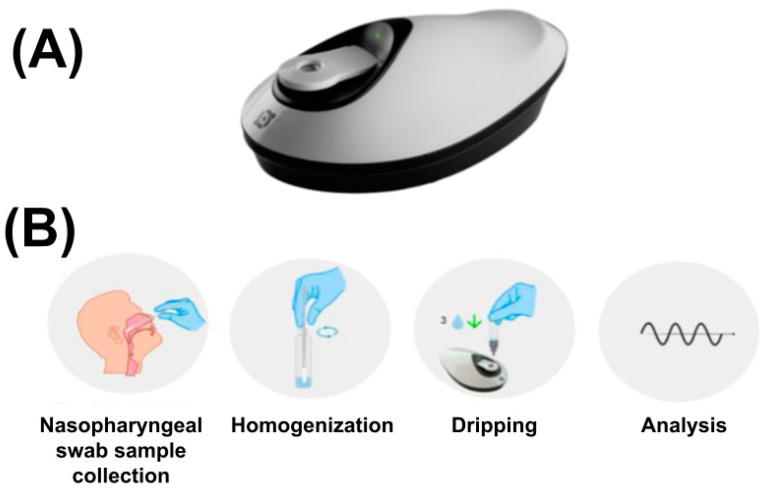
(**A**) Hilab Volt. (**B**) COVID-19 sample collection and analysis procedure.

With the aim of increasingly democratizing access to health, in 2017, Hilab was born. All of our devices are developed in-house by multidisciplinary researchers and the Hilab Volt (ANVISA Register n° 80583710019, Figure 1A) is the newest generation of Hilab devices, which is based on electrochemical techniques aimed at clinical diagnosis. Electrochemical platforms meet the POC requirements, thus reaching a larger portion of the population and further democratizing access to healthcare [4,5]. 

Electrochemical sensors have unique potential properties that are strongly related to their high sensitivity, selectivity, and stability, making them widely used in environmental, industrial, and medical fields. Recent evidence demonstrates that electrochemical technology provides a promising platform for primary healthcare, primarily through real-time monitoring [6]. To improve the response presented by this class of sensors, superficial modification has been used. The modification increases the interaction capacity through the use of biomolecules that selectively recognize the analyte, such as enzymes and antibodies, increasing the specificity of electrochemical sensors. Furthermore, by changing the surface chemistry, it is possible to increase both the surface reactivity and its area, which directly implies an improvement in the sensitivity of these tests [7].

In the COVID-19 pandemic scenario, some electrochemical options are described in the literature for the determination of both the virus and the antibodies [8,9,10], in which superficial modification with labeled target analytes or secondary antibodies is used. These are undoubtedly powerful and robust methodologies with wide applicability, but despite the good characteristics presented by this class of sensor, there are significant issues associated with the labeling process, the probe use and dependence, and the inherently multi-step nature undesirable for POC applications. Label-free modification approaches benefit considerably from being potentially single-step, fast, and inexpensive, but unfortunately, it is not uncommon that the reliance on transduction in a single binding process brings with it profound problems associated with the nonspecific response [11]. There is, nonetheless, a significant and growing body of work showing that high levels of selectivity can be achieved through controlled interfacial chemistry allied with an unusual technique based on non-faradaic processes.

In general, electrochemical impedance spectroscopy (EIS) can be low cost, low-power-demanding, and highly sensitive, with minimal hardware requirements. Like all electrochemical approaches, the scalability, multiplexing, and miniaturization are strong positive points [12]. By imposing potential sweeps or steps, the electrochemical cell is driven to a condition far from chemical equilibrium, and a transient response signal associated with the non-equilibrium state is observed [13,14,15]. Non-faradaic electrochemical impedance spectroscopy (NF-EIS) is a powerful technique for capturing subtle changes in the binding interaction at the electrode–solution interface without the need for a redox molecule [14,15] and can provide the rapid and sensitive detection of biomarkers and analytes. These advances will generate diagnostic methods based on NF-EIS, potentially being better for healthcare applications [15].

In this work, we describe a new single-step, fast, low-cost, point-of-care technology that produces results in 5 min. The test can be performed at room temperature with minimal equipment and reagents, being carried out directly on the nasopharyngeal sample collected, without any pre-treatments. The most important aspects and experimental conditions of the technique and sensing system are first determined, allowing the early diagnosis.

## 2. Materials and Methods

### 2.1. Materials

The reagents were of analytical grade and were used without further purification. Sodium phosphate monobasic (NaH_2_PO_4_) and anhydrous sodium phosphate dibasic (Na_2_HPO_4_) were obtained from Êxodo Científica (Sumaré, São Paulo, Brazil). Tween-20 (TW-20), Bovine Serum Albumin (Lyophilized powder > 96%, agarose gel electrophoresis-BSA), D-(+)-Trehalose dihydrate, and Proclin^®^300 were purchased from Sigma-Aldrich (St. Louis, MO, USA). Saccharose was obtained from Cloroquímica (Curitiba, Paraná, Brazil). High-purity deionized water (resistivity of 18.2 MΩcm) was obtained from a Milli-Q system (Millipore, Burlington, MA, USA). The experiments were performed at room temperature (~25 °C). Purified SARS-CoV-2 nucleocapsid protein (FPZ0513; SC2-NProt) was obtained from Fapon (Taiwan, China). The SARS-CoV-2 antigen sample control (0810590CFHI) was obtained from Zeptometrix. Influenza A (INF A, AGFLU-03), Influenza B (INF B, AGFLU-10), and Respiratory Syncytial Virus (RSV, ATG-122) control samples were obtained from Controllab Controle de Qualidade para Laboratórios LTDA (Rio de Janeiro, Rio de Janeiro, Brazil). Proprietary PET-based screen-printed carbon electrodes were used for the immunosensor construction. The working (WE; with a geometric area of 7.07 mm^2^) and counter (CE) electrodes were carbon-based, and the pseudo-reference electrode (pRE) was silver/silver chloride.

### 2.2. Chemical and Sensing Interface Preparation

The conjugation step was mainly performed as previously described [16]. Briefly, 40 nm colloidal gold nanoparticles were passively conjugated to commercially available SARS-CoV-2 anti-nucleocapsid antibodies (Arista Biologicals, Allentown, PA, USA) after screening for the best pH and concentrations to stabilize them [17,18]. The stock colloidal gold conjugate (CGC) concentration was 10 OD, and after the conjugation, the CGC was diluted to the optimal 2-OD concentration (2OD-CGC) using a 0.01 M 7.4-phosphate buffer solution (7.4-PB) containing saccharose, trehalose, BSA and TW-20 (1:1:0.2:0.01% *w*/*w*). The new OD was confirmed with a Nanodrop One UV-Vis Spectrophotometer at 530 nm. For the immunosensing interface preparation, 2.0 μL of freshly prepared 2OD-CGC solution was dropped on the WE surface and dried for 30 min at room temperature (CovAg-SPE). 

### 2.3. Electrochemical Measurements and Optimization

All the electrochemical measurements were carried out using the Hilab Volt (Electrochemical Reader; ANVISA Register n° 80583710019). To assess the performance of the immunosensor, electrochemical impedance spectroscopy (EIS) was used for the method development, using the fixed frequency mode. A signal composed of both DC and AC was used to polarize the interface, equal to 0.0 and 10.0 mV, respectively. The fixed frequency was 100 Hz, in a logarithmic scale with 1 point per 10 s, for 300 s. 

All the measurements were carried out in pH 7.4-PB (0.01 M) using Proclin 0.05% as a preservative agent. The analytical NF-EIS experiments were carried out using the proper SC2-NProt dilution dropped directly on the CovAg-SPE surface. 

### 2.4. Clinical Application

Human nasopharyngeal swab samples were collected in duplicate from a local hospital and were characterized by RT-PCR. These samples (N = 252) were collected in the universal transport media (UTM, for reverse transcription polymerase chain reaction analysis-RT-PCR) or in developed media (for electrochemical analysis), stored under refrigeration (2 to 8 °C) until the moment of transport, and transported to the company daily, so that the analysis could be performed on fresh samples. All the sample testing was performed using portable devices in a bio-safe environment, and the analytical team was blinded to the clinical and/or personal information of the patients and the samples. The Research Ethics Committee of Pontifícia Universidade Católica do Paraná-PUC/PR approved this study (53317121.0.0000.0020), and all the methods were performed following the relevant guidelines and regulations. Written informed consent has been obtained from the volunteers to publish this paper. Figure 1B shows the collection and analysis procedure.

## 3. Results and Discussion

### 3.1. Impedimetric Measurement

For an EIS-based biosensor, the detection signal can be obtained either from faradaic or non-faradaic processes, which occur at the electrodes due to selective bindings or interactions with the recognition layer [19]. Each of the proposals presents advantages and disadvantages concerning their applications, but taking into account the application as a POC device and a methodology in which the smallest number of steps is the most adequate, a non-faradaic methodology was developed due to the simplicity of the measurement process.

**Figure 2:**
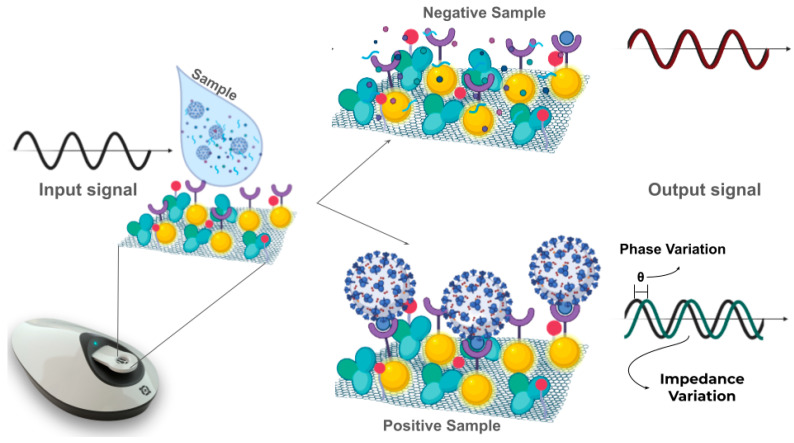
Proposed COVID-19 diagnostics principle for the CovAg-SPE sensor analyzing negative and positive nasopharyngeal swab samples, and the parameters influencing the input and output signals.

The proposed procedure consists of only one step, in which the previously collected sample is dripped on the CovAg-SPE surface. Shortly thereafter, the equipment is activated and the data collection regarding the interaction between the sample and the selective layer is carried out as a function of the time. Figure 2 exemplifies the process. It is necessary to emphasize that to the best of our knowledge, this procedure is not common in the literature for biosensors or POC devices yet, especially outside of flow-state measurements [15], but this is an invaluable characteristic of the methodology that can be further explored. The single-step measurement in the sample condition greatly facilitates the usability of the sensor for POC purposes.

The addition of a positive sample, i.e., that interacts with the electrode surface, will result in a different profile than the profile observed for negative samples. So, by creating and optimizing a selective layer on the electrode, it was possible to identify an analyte in the sample (protein or antibody) and to differentiate positive and negative samples by the resulting NF-EIS profile [15]. For each sample analyzed, two major characteristic signatures are observed based on the imaginary impedance (Zi) and phase (θ) values, and from this, the samples can be sorted as positive or negative, with the aid of artificial intelligence and machine-learning (ML) techniques. 

Two frequency bands were evaluated (Figure S1) that could be useful for differentiating samples, 100 and 1000 Hz, both with positive and negative samples and as observed in the literature [20,21]. The results showed a significant difference in the impedance variation observed at a lower frequency, that is, using 100 Hz instead of 1000 Hz, making it possible to better differentiate (positive/negative ratio >1) the samples when a lower frequency is used. The behavior observed and described above, with more significant variations at a low frequency, follows impedimetric sensors, in which the non-faradaic behavior is predominant since in these cases the observed response is mostly governed by the capacitive behavior of the double electrical layer [21]. The addition of a positive sample and interaction with the N-PROT provides another contribution to the capacitance (and resulting impedance), as it is a non-conductive layer. The differentiation is possible since the behavior of the sensors against positive and negative samples is different, and this difference is reflected in the values of Zi and θ presented for each group of samples.

### 3.2. Chemical Optimization and Analytical Performance

To obtain the best performance from the developed sensor, some parameters were optimized. Firstly, the composition of the dilution solution and CGC concentration were studied, seeking conjugate stability and better differentiation of positive and negative samples. 

The dilution solution was optimized as saccharose, trehalose, BSA, and TW-20 (1:1:0.2:0.01% *w*/*w*), diluted in 0.01 mol L^–1^ pH 7.4 phosphate buffer solution. Saccharose and trehalose were added for stability purposes, increasing the shelf-life expectancy of the modified sensor by creating a glass-like layer that protects the conjugate from contact with the exterior of the modification [22,23,24]. The BSA was added to limit the quantity of unspecific reactions between the conjugate and the complex samples during the measurement [25,26]. The CGC was diluted to an optimal concentration of 2OD (Figure S2A) using the dilution solution, based on the sensor performance and differentiation between negative- and positive-spiked samples. The modification was also studied, with an optimized volume of 2.0 µL of 2OD-CGC being used to modify the WE area.

It is well known that capacitive biosensors are less sensitive than faradaic biosensors [14,27,28], and a well-applied approach to enhancing this is to decrease the ionic strength of the solution used in the EIS measurements. Thus, a concentration of PBS buffer lower than the usual was used (0.01 mol L^–1^), as the capacitance change upon target binding becomes more relevant at lower frequencies (Figure S2B).

**Figure 3:**
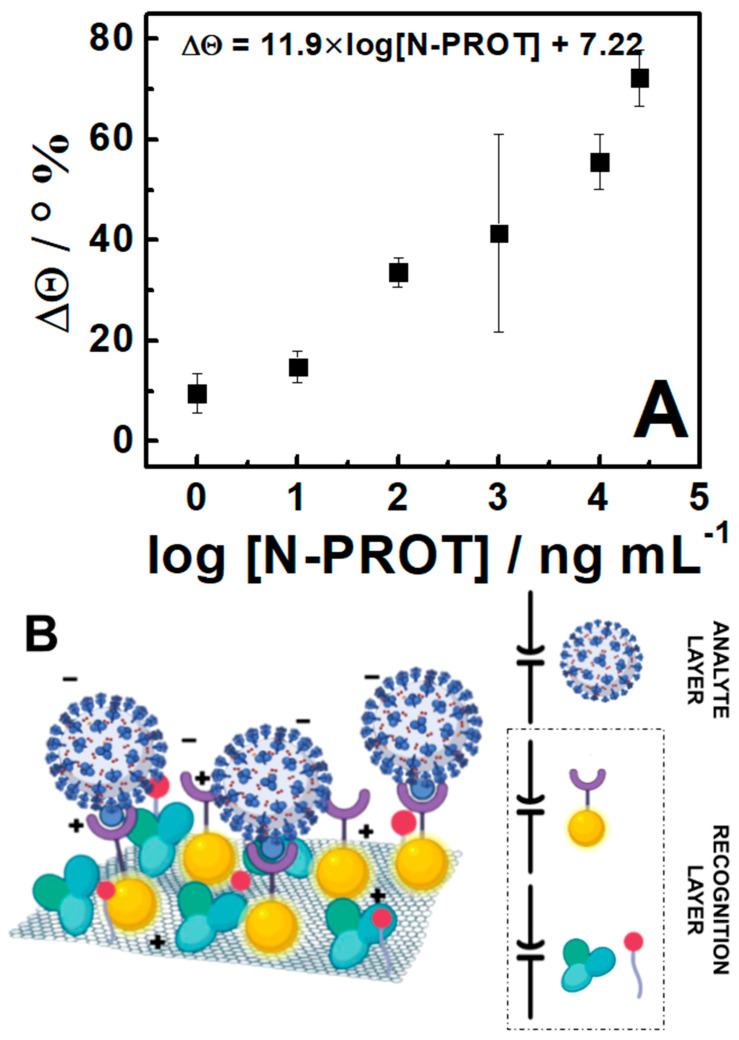
(**A**) Electrochemical calibration curve for N−protein detection in spiked negative nasopharyngeal swab samples (*n* = 3). (**B**) Schematic representation of the superficial interactions and capacitors formed by the recognized layer and the analyte layer. t = 300 s; f = 100 Hz; EAC = 0.01 V; EDC = 0 V; electrolyte: 0.01 M 7.4−PB.

The analytical performance of the developed sensor was obtained using the impedance response from several N-PROT-spiked swab solutions, obtained from negative samples, in different ranges of concentration. The impedance was measured at a frequency of 100 Hz, during 300 s, for *n* = 3. The results are plotted as the varying signal of the phase (in % of the shift in relation to blank θ°) versus the N-PROT in the range from 1.0 ng mL^–1^ to 25 μg mL^–1^, as shown in Figure 3. It is possible to observe that there is a correlation of log-linear variation between the phase and the increase in the protein concentration in the fortified samples, in the range of 1.0 ng mL^–1^ to 10.0 μg mL^–1^, described for Δθ = 11.9 ± 3.1*log [N-PROT] + 7.22 ± 1.4. However, it should be noted that the protein availability for interaction at the electrode is contained in the range of 0.15 ng to 1.5 μg, since volumes of 150.0 μL were used. Table 1 shows the comparison with other works described in the literature for SARS-CoV-2 antigen detection using electrochemical devices.

Studies investigating the correlation between the SARS-CoV-2 nucleocapsid antigen test positivity and the transmissible window of COVID-19 have recently been published, revealing that patients with positive nucleocapsid antigen tests had a shorter duration of viral shedding and a shorter transmissible window of COVID-19 compared to those with positive PCR tests. They also suggest that the nucleocapsid antigen test may be particularly useful in identifying individuals who are most likely to transmit the virus, as the tests had high sensitivity during the first week of illness when the viral load is highest and individuals are most infectious. Overall, these studies suggest that nucleocapsid COVID-19 antigen tests may be a useful tool for identifying individuals who are most likely to transmit the virus and for determining the duration of the transmissible window of COVID-19 [29,30,31].

With this in mind, and considering that the approximate limit of detection of N-protein in commercially available lateral flow COVID-19 antigen tests ranges from 2.0 to 6.0 ng mL^–1^ [16], the calculated LOD for our electrode (1.0 ng mL^–1^; 3×SD(blank)/slope) does not represent a significant decrease in the clinical sensitivity of the test in infected patients, who usually show a much higher concentration than this, but rather establishes our solution as an interesting alternative to traditional lateral flow assays for mass SARS-CoV-2 testing and isolation of transmissible individuals. So, when the LODs are compared, the proposed device presents a higher value when compared to the most described devices but is the only device that does not need an incubation time, plus a washing step and the measurement time, which facilitates all the operation and performance of the test. 

The developed sensor relies on a completely different approach as compared to the other electrochemical tests described in Table 1 and several other commercially available antigen tests for SARS-CoV-2, which could represent a valuable alternative as a portable diagnostic platform for the rapid screening for COVID-19, opening up an opportunity for the development of a device for other easily transmissible viral diseases.

The repeatability and reproducibility (Figure S3) study was carried out using two sample levels, reagent and non-reagent, being tested ten times in a row for each level. It can be observed that the variations in the sensors, from the average value, do not exceed 10%. Furthermore, accuracy percentages >99% were obtained for both levels.

The stability study (Figure S4) was conducted for three months, during which it was possible to observe differentiation between positive and negative samples (positive/negative ratio >1).

### 3.3. AI-Based Classification and Clinical Performance

The analysis procedure proposed in this project is carried out at a fixed frequency, with the impedance results being registered as a function of the time, in which the differentiation between positive and negative samples is performed based on the variation (delta) in the system phase (θ) and imaginary impedance (Zi) through measurement. Thus, within the 5 min of single-frequency real-time EIS measurement, it was observed that the positive samples had a significantly greater impedance and phase variation than the negative samples. 

**Figure 4:**
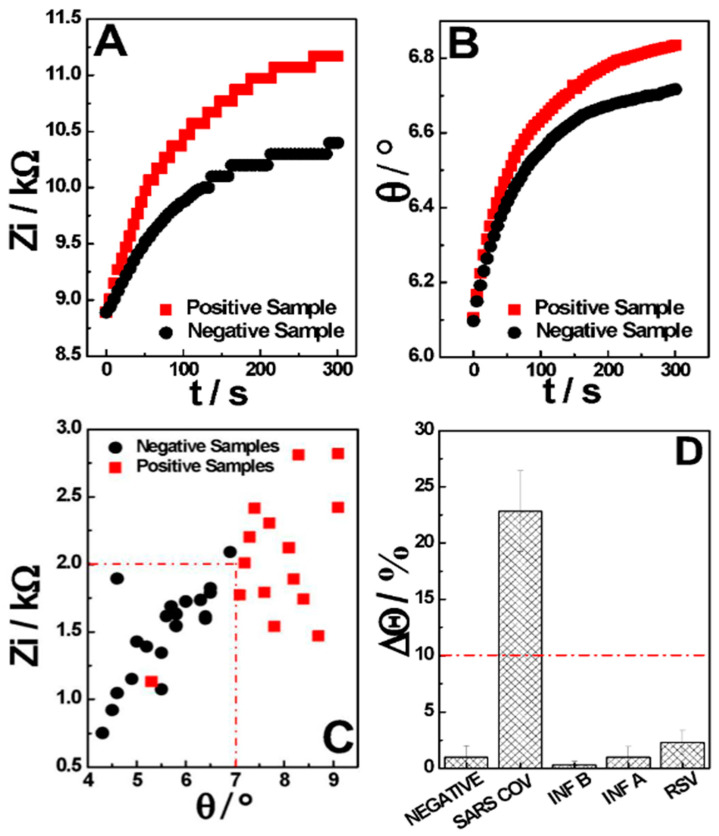
Positive and negative sample behavior as a time function for (**A**) imaginary impedance, and (**B**) phase. (**C**) Characteristic behavior used for classification of real samples in validation. (**D**) Bar graph constructed from the concomitant species study. t = 300 s; f = 100 Hz; EAC = 0.01 V; EDC = 0 V; electrolyte: 0.01M 7.4-PB.

The majority of the negative samples present a variation in Zi smaller than 1.8 kΩ, with a phase variation smaller than 7.0 ° (Figure 4A,B, in black). On the other hand, most of the positive samples show a variation in Zi above 2.0 kΩ, with a phase variation above 7.0 ° (Figure 4A,B, in red). It was noted that the imaginary impedance contribution to the total impedance change was higher than the real impedance contribution, leading to a higher differentiation between positive and negative samples. This reflects the measurement conditions, where no electrochemical probe is present, and the capacitive contributions of the interaction between the conjugate and the N-PROT play a major role in the total impedance shift. Thus, using the two factors, the phase and the impedance imaginary components (Figure 4C), the samples can be classified through AI, detecting when both cut-off values are surpassed and indicating if a sample is negative or positive (Figure 4D). In a nutshell, each of these factors is not able to differentiate samples efficiently on its own, as can be seen in Figure S5, which shows the ROC curve obtained for each factor, but considering the two or more, there is a clear threshold where the diagnostic becomes possible. The ML process helps with the cut-off definition, where new sample data with known diagnostic information can be fed to the system in order to decrease the error in the sample differentiation, sharpening the method with its use. From this, the samples are diagnosed with an appropriate regression model to predict if they are SARS-CoV-2 positive or negative. As a result, our model plots a graph between the variables that best fit the given data points (Figure S6) and returns to us the probability of diagnosis. 

Based on these specifications, it was possible to analyze and classify the 252 samples collected. The human nasopharyngeal swab samples were also analyzed by the real-time PCR method to confirm the presence of SARS-CoV-2 and compared with the obtained results. A confusion matrix, which demonstrates the classification of data obtained during the validation stage considering all 252 analyzed samples, was constructed and is described in Table 2. From the data, it is possible to observe that the developed device presents a strong correlation between the positive samples flagged by the AI and the diagnostic provided by the gold standard for such analyses. Also, the values obtained for the sensitivity, specificity, and accuracy, as well as the confidence intervals (CIs) for each data (Table 3), were considered quite satisfactory, given that the results were acquired within 5 min and without sample processing. From the statistical Kappa (κ) test, performed to analyze the interrater reliability of the qualitative measurements, a κ coefficient of 0.81 was obtained, which is statistically significantly different from zero, implying a strong agreement (κ > 0.81) (Table 4, [32]). Therefore, there is a clear comparison between behavioral and sample discrimination.

**Table 1:** Comparison between devices described for SarsCov-2 N-protein determination.

Sensor	Sample	Electrochemical Feature	LOD/ng mL^−1^	Time Required	Ref
Aptasensing nucleocapsid protein on nanodiamond-assembled gold-interdigitated	Nasopharyngeal	Redox probe solution using EIS	1.82 × 10^−5^	5 min ^1^ + Measurement	[33]
Anti-N on MUA-AuNPs-modified SPE	Nasopharyngeal	Redox probe detection using SWV	0.0004	15 min ^1^ + Measurement	[34]
N-protein on carbon nanofiber-modified SPE	Nasopharyngeal	Competitive redox probe detection using SWV	0.0008	20 min ^1^ + Measurement	[35]
Magnetic bead-based immunosensor combined with carbon black-modified screen-printed electrode	Saliva	Magnetic beads and alkaline phosphatase labeled using DPV	8.0	30 min ^1^ + Measurement	[36]
Anti-N on screen-printed gold electrodes assisted by labeled magnetic beads	Serum	Redox probe amperometric detection	0.23	20 min ^1^ + Measurement	[37]
N-protein molecularly imprinted polymer	Nasopharyngeal	Redox probe detection using DPV	0.0007	15 min ^1^ + Measurement	[38]
Anti-N conjugated with AuNPs/Sac/Tre/SPE (CovAg-SPE)	Nasopharyngeal	Capacitive measurement using EIS	1.0	5 min	*

* This work; SPE: Screen-printed electrode; NP: Nanoparticles; MUA: 11-Mercaptoundecanoic acid; EIS: Electrochemical impedance spectroscopy; SWV: Square-wave voltammetry; DPV: Differential pulse voltammetry. ^1^ Incubation time.

The specificity of this immunosensor was evaluated by performing measurements of control samples of the most common respiratory viruses that usually cause COVID-19-like symptoms: Influenza A (INF A), Influenza B (INF B), and Respiratory Syncytial Virus (RSV). As can be seen in Figure 4D, the signal response of the developed sensor was considerably higher against SARS-CoV-2 (variations greater than 10% when compared to the signal of negative samples) presence as compared to the responses against the possible interfering viruses, suggesting that these concomitant species do not cross-react with this sensor, thus demonstrating the appreciable selectivity of the proposed device.

## 4. Conclusions

In this work, a single-step, rapid, low-cost, and point-of-care technology that produces qualitative and quantitative results within 5 min was developed by our group. Our test can be performed at room temperature with minimal equipment and reagents. The sensor configuration and the transduction technique demonstrated the capability to detect N-protein in the directed collected nasopharyngeal samples, without any pre-treatments. Also, advantages such as not needing incubation and cleaning steps, which reduces the total steps and cost, no labor procedures due to the direct real-time label-free detection, and a POC system that allows it to be used remotely and in a decentralized laboratory environment were achieved. 

To show the proper functionality and applicability of the N-PROT sensor for COVID-19 diagnosis, measurements of 252 clinical nasopharyngeal swab samples were demonstrated. Comparison against the gold standard procedure successfully validated the correct distinction between healthy patients and patients infected with SARS-CoV-2. The Kappa concordance test presented a strong correlation between the methodologies (k = 0.81), and 83.0, 96.2, and 91.3% sensitivity, specificity, and accuracy were achieved, respectively. In addition, a remarkable relationship was observed between the days of symptoms and CT, in which the best results were obtained with samples from patients with at least 3 days of symptoms and CT of 35 or less.

Normally, label-free electrochemical sensors are not the first option when an application need arises due to the disadvantages presented by this class of sensors when compared to labeled sensors. However, recent advances in instrumentation have made label-free sensor systems sensitive enough to characterize the changes on the electrode surface, allowing real-time monitoring of the analyte concentrations in different matrices, making this class of sensors serve as the focus of development in recent years.

This class of sensors can be easily applied to methodologies involving antigen–antibody immunoreactions, enabling the development of platforms for the detection of a range of analytes, from tumor markers to neglected diseases.

## Figures and Tables

**Table 2:** Confusion matrix obtained for the clinical samples analyzed in the validation of collected data.

		Gold Standard		Total
		+	−	
Hilab	+	78	6	84
	−	16	152	168
Total		94	158	252

**Table 3:** Analytical parameters obtained from the confusion matrix validation data.

		CI/%
Sensitivity	83.0%	74.0—89.0%
Specificity	96.2%	92.0—98.0%
Accuracy	91.3%	86.4—100.0%
PPV ^1^	92.9%	-
NPV ^2^	90.5%	-

^1^ Positive predictive value; ^2^ Negative predictive value.

**Table 4:** Results obtained for the Kappa concordance test.

p0 ^1^	0.91
pe ^2^	0.54
SE(k)	0.04
k	0.81
CI	0.73 to 0.89
Strong Agreement	

^1^ p0: Relative acceptance rate; ^2^ pe: Hypothetical acceptance rate.

## Data Availability

The data that support the findings of this study are available from the corresponding author, A. Gevaerd, upon reasonable request.

## Supplementary Materials

The following supporting information can be downloaded at https://www.mdpi.com/article/10.3390/s24123772/s1, Figure S1. Results concerning the function of the positive/negative ratio in the frequency study. t = 300 s; EAC = 0.01 V; EDC = 0 V; electrolyte: 0.01M 7.4-PB. Figure S2. Results concerning the function of the positive/negative ratio obtained for A) the CGC dilution study and B) the ionic strength of buffer solution study. t = 300 s; f = 100 Hz; EAC = 0.01 V; EDC = 0 V; electrolyte 7.4-PB. Figure S3. Results obtained for the precision study (repeatability and reproducibility) for the A) non-reagent and B) reagent samples. t = 300 s; f = 100 Hz; EAC = 0.01 V; EDC = 0 V; electrolyte: 0.01M 7.4-PB. Figure S4. Results concerning the function of the positive/negative ratio in the stability study. t = 300 s; f = 100 Hz; EAC = 0.01 V; EDC = 0 V; electrolyte: 0.01M 7.4-PB. Figure S5. ROC curves for the detection of positive and negative samples based on the A) phase and B) imaginary impedance variation. t = 300 s; f = 100 Hz; EAC = 0.01 V; EDC = 0 V; electrolyte: 0.01M 7.4-PB. Figure S6. Graphical result obtained from the developed predictive machine-learning algorithm for classifying the sensor response data.
