# Supplementary material for: Utilizing COVID-19 as a Model for Diagnostics Using an Electrochemical Sensor
Source: Sensors (Basel). 2024 Jun 10;24(12):3772. doi: 10.3390/s24123772 (PMC11207896; doi:10.3390/s24123772)

Supplementary Materials

Utilizing COVID-19 as a Model for Diagnostics Using an Electrochemical Sensor

Ava Gevaerd [1,*], Emmanuelle A. Carneiro [1], Jeferson L. Gogola [1], Diego R. P. Nicollete [1], Erika B. Santiago [1], Halanna P. Riedi [1], Adriano Timm [1], João V. Predebon [1], Luis F. Hartmann [1], Victor H. A. Ribeiro [1], Carlos Rochitti [2], Gustavo L. Marques [2], Maira M. O. N. Loesch [2], Bernardo M. M. de Almeida [1], Sérgio Rogal-Junior [1] and Marcus V. M. Figueredo [1]

[1] Research and Development Department, Hilab Campus, Rua José A. Possebom, 800, Curitiba 81270-185, Parana, Brazil; marcus@hilab.com.br (M.V.M.F.)
[2] School of Medicine—Campus PUCPR, Rua Imaculada Conceição, 1155, Prado Velho, Curitiba 80215-901, Parana, Brazil
* Correspondence: ava.gevaerd@hitechnologies.com.br

Figure S1. Results concerning the function of the positive/negative ratio in the frequency study. t = 300 s; E_{AC} = 0.01 V; E_{DC} = 0 V; electrolyte: 0.01M 7.4-PB.

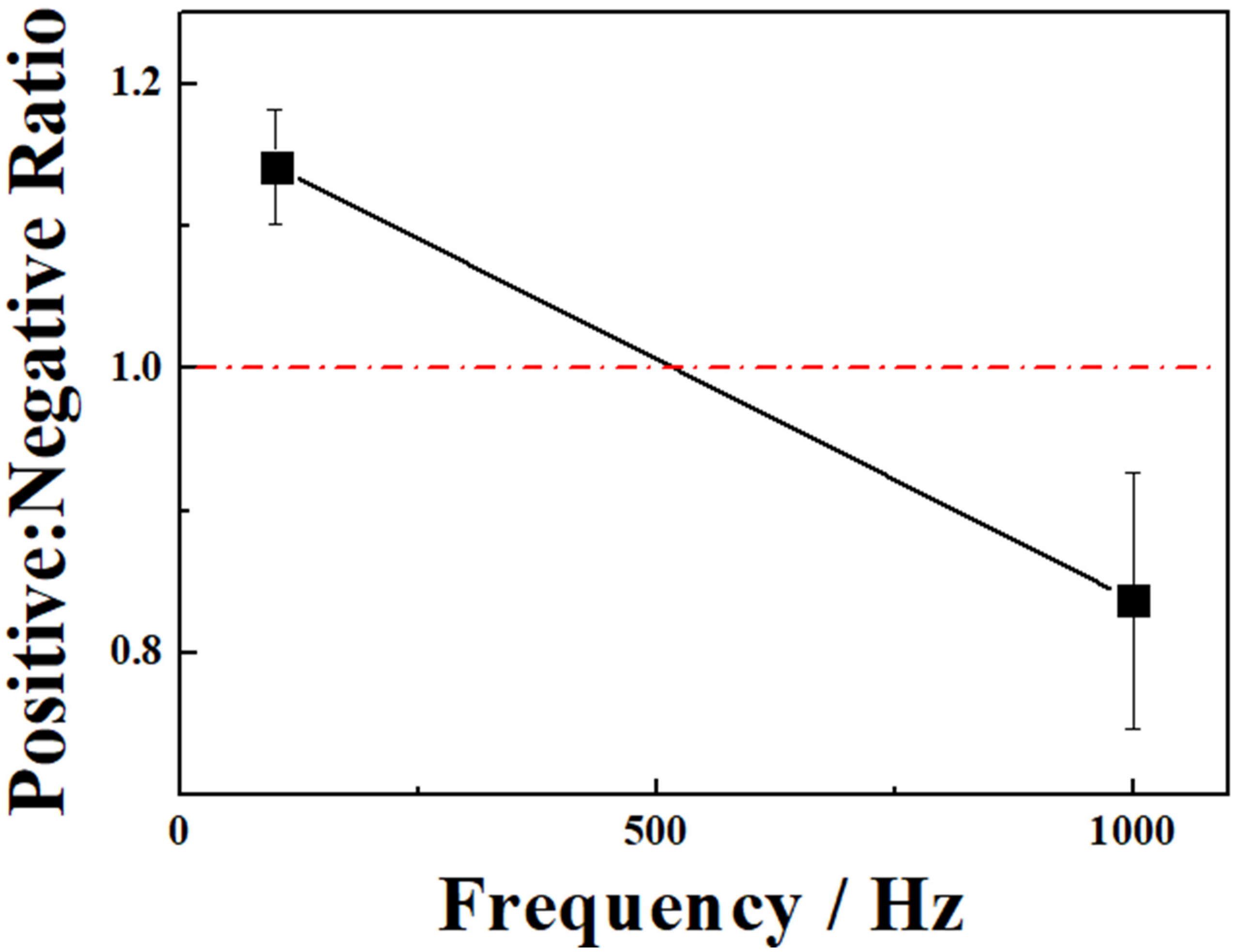

Figure S2. Results concerning the function of the positive/negative ratio obtained for A) the CGC dilution study and B) the ionic strength of the buffer solution study. t = 300 s; f = 100 Hz; E_{AC} = 0.01 V; E_{DC} = 0 V; electrolyte 7.4-PB.

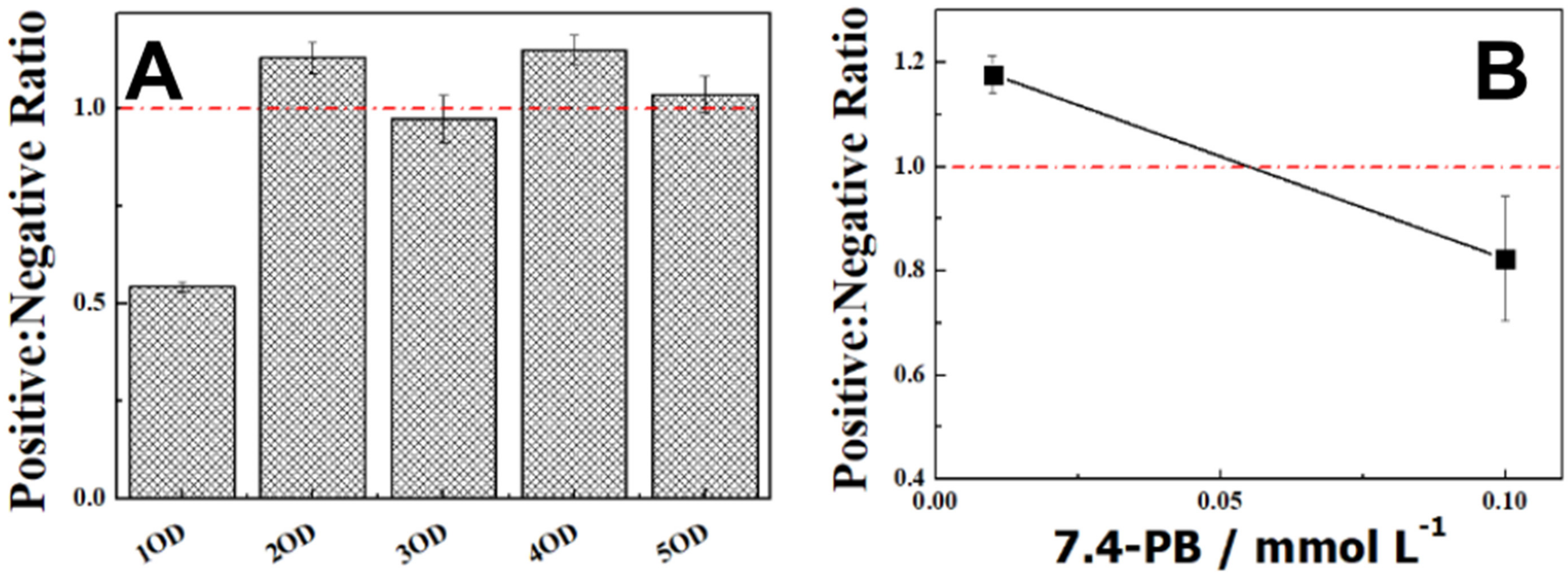

Figure S3. Results obtained for the precision study (repeatability and reproducibility) for the A) non-reagent and B) reagent samples. t = 300 s; f = 100 Hz; E_{AC} = 0.01 V; E_{DC} = 0 V; electrolyte: 0.01M 7.4-PB.

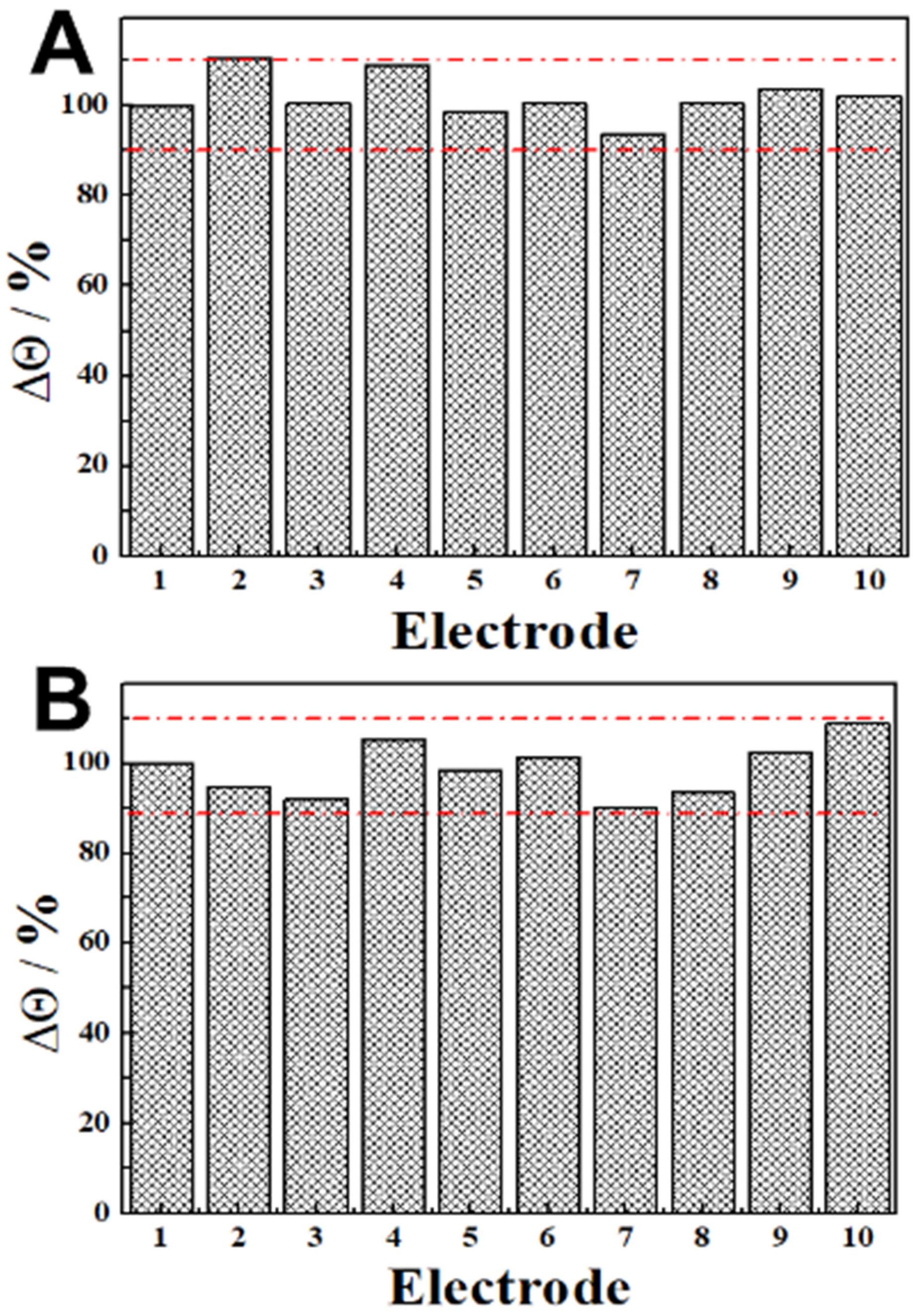

Figure S4. Results concerning the function of the positive/negative ratio in the stability study. t = 300 s; f = 100 Hz; E_{AC} = 0.01 V; E_{DC} = 0 V; electrolyte: 0.01M 7.4-PB.

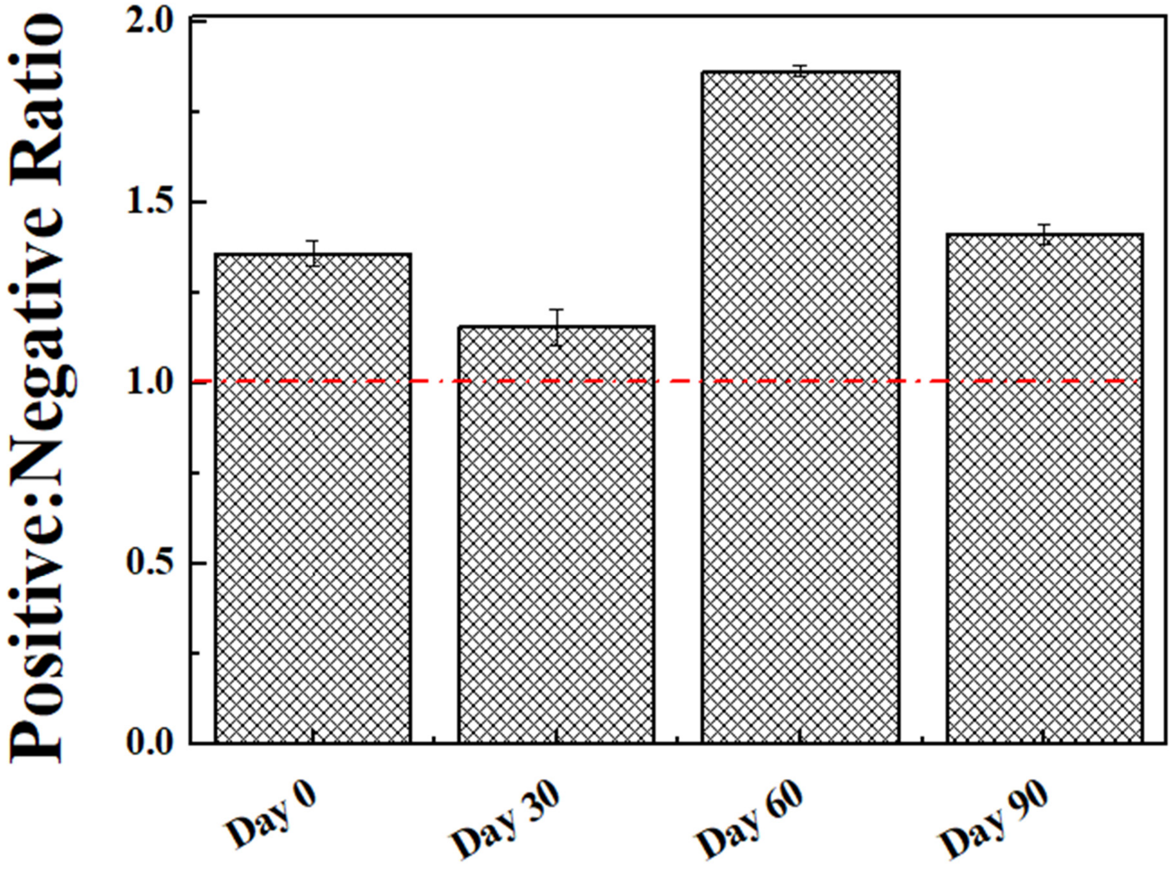

Figure S5. ROC curves for the detection of positive and negative samples based on the **A)** phase and **B)** imaginary impedance variation. t = 300 s; f = 100 Hz; E_{AC} = 0.01 V; E_{DC} = 0 V; electrolyte: 0.01M 7.4-PB.

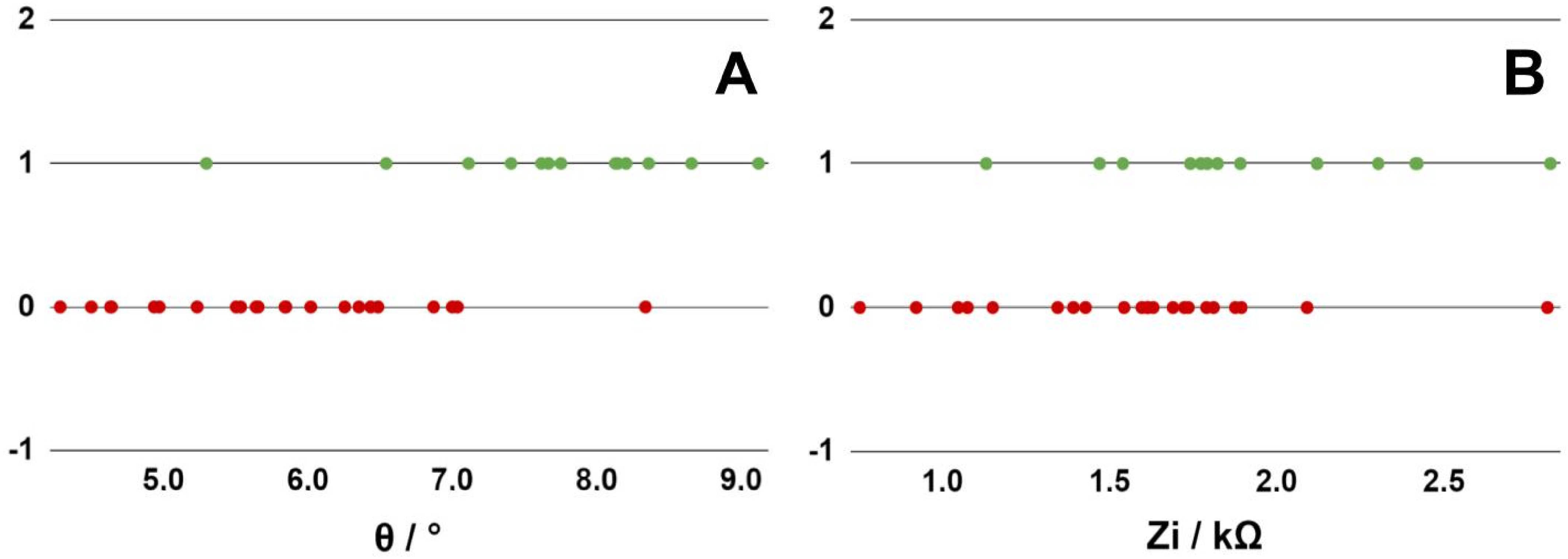

Figure S6. Graphical result obtained from the developed predictive machine-learning algorithm for classifying the sensor response data.

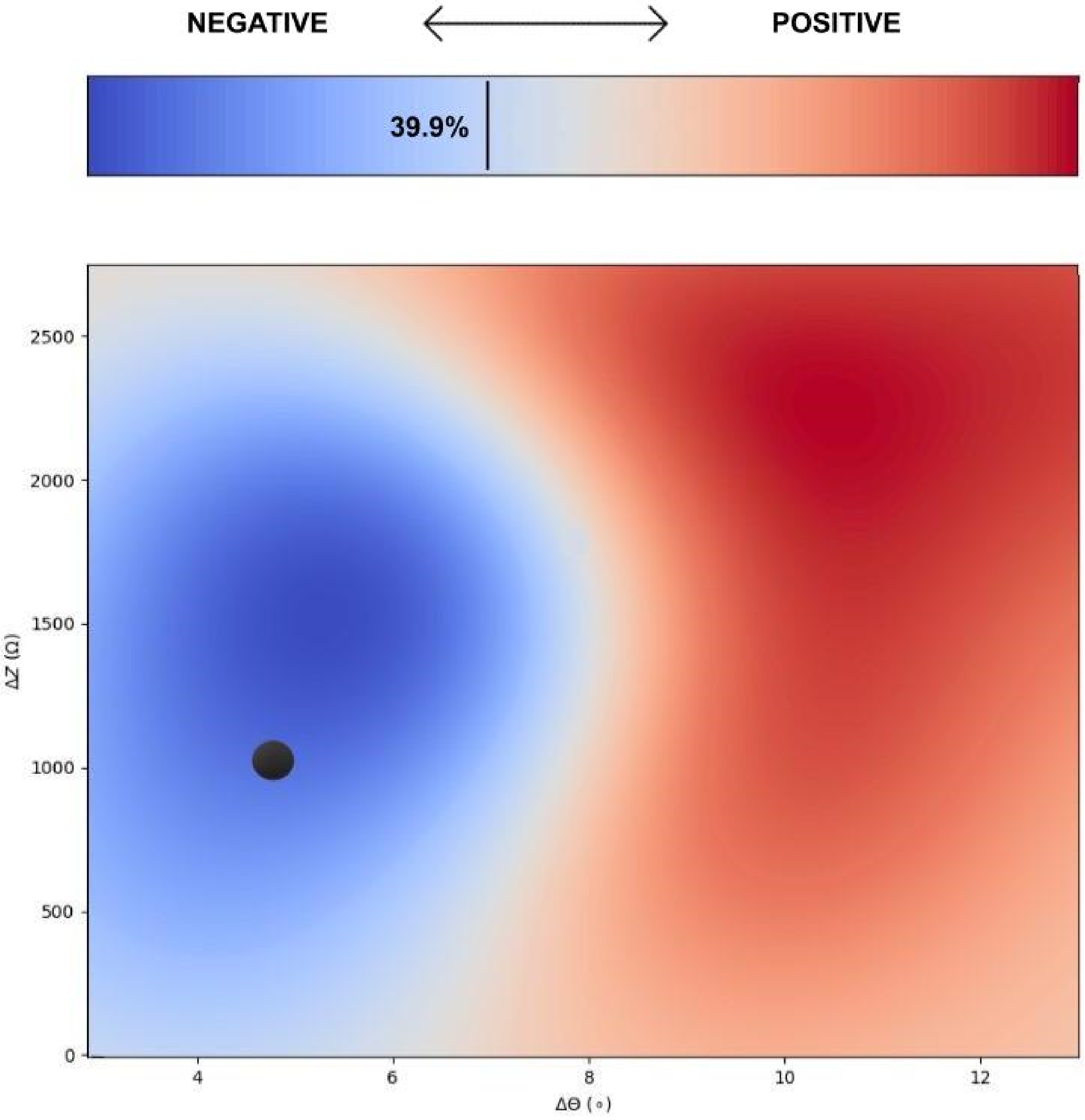

Supplement: Supplementary file 1 [file sensors-24-03772-s001.zip › sensors-3022267-supplementary.pdf]
